# Feral pigs as a reservoir for zoonotic and transboundary diseases in the Western Pacific Region

**DOI:** 10.5365/wpsar.2024.15.1.1114

**Published:** 2024-03-29

**Authors:** Andrew M Adamu, Cadhla Firth, Bruce Gummow, Roslyn I Hickson, Andrew J Hoskins, Paul F Horwood

**Affiliations:** a College of Public Health, Medical and Veterinary Sciences, James Cook University, Townsville, Queensland, Australia.; b Australian Institute of Tropical Health and Medicine, James Cook University, Townsville, Queensland, Australia.; c EcoHealth Alliance, New York City, New York, United States of America.; d Commonwealth Scientific Industrial Research Organisation, Townsville, Queensland, Australia.

Over the last century, the global human population has more than quadrupled, leading to anthropogenic and environmental impacts that have disrupted the interactions between pathogens, humans and animal hosts. More than 60% of emerging infectious diseases affecting humans are considered zoonotic, with > 70% of these originating from wild animals. (*1*)

Feral pigs (*Sus scrofa*) are one of the most prevalent invasive species worldwide, with an estimated population size of over half a billion. (*2*) Feral pigs are regarded as a “triple threat pest” due to: 1) their propensity to be reservoirs for important transboundary diseases; 2) the serious threats they pose to native flora and fauna; and 3) the massive impacts they have on agricultural production and practices. (*3*) The rapid reproduction rate of feral pigs, their omnivorous, flexible and opportunistic diet, the absence of predators in many areas, and their ability to thrive in anthropogenic landscapes are all factors driving ongoing expansion of their geographic range. (*4*) Human activities are key to the expansion of the feral pig population, as anthropogenic modifications to the ecosystems that support feral pigs can boost population densities over the natural carrying capacity.

To date, limited research or monitoring activities have been conducted to determine the risk that feral pigs pose as reservoirs for zoonotic or veterinary pathogens. As the number of feral pigs increases, they encroach into human habitats in search of feed, thus amplifying the risk of zoonotic disease transmission. (*4*) Feral pig hunters, farmers, slaughterhouse workers and animal health workers are at increased risk of contracting zoonotic diseases associated with feral pigs. In a study from northern Australia, 90% of brucellosis cases were reported in feral pig hunters. (*5*) More broadly in the Western Pacific Region, other emerging threats associated with feral pigs include Japanese encephalitis (JE), African swine fever (ASF), foot and mouth disease (FMD), influenza A and Nipah viruses. In addition, feral pigs may act as reservoirs for endemic pathogens with possible impacts on human and livestock health such as hepatitis E virus, *Coxiella burnetii* (Q fever), *Brucella suis* (brucellosis), *Streptococcus suis* and *Leptospira spp.* (leptospirosis). In the Western Pacific Region, feral pigs are widely distributed and are speculated to play a key role in the maintenance and spread of several pathogens of international concern. Other pig species are also found in the Western Pacific (such as the threatened *Sus barbatus* and *Porcula salvania*), but not much is known about the threats to conservation due to the diseases carried by feral pigs.

The recent emergence of JE in mainland Australia (February 2022) has highlighted the risk that once the virus has been introduced, feral pigs may act as an amplifier and/or reservoir host for the establishment of the virus in new geographic areas. (*6*) JE is a complex zoonotic transboundary arboviral infection involving both vertebrates (pigs, birds) as reservoirs/amplifying hosts and arthropod vectors (*Culex* mosquitoes). (*7*) Recent modelling studies have highlighted the role of feral and domestic pigs in the epizootic and epidemic risk of JE in the natural cycle of transmission in Australia. (*6*) Studies in other settings have reported pig-to-pig transmission (non-vector transmission), mediated by the high replication of the JE virus in swine tonsils, allowing oronasal spread between animals, implying higher risks of enzootic establishment associated with pig populations. (*8*)

ASF is an arboviral haemorrhagic disease caused by the ASF virus, which can be transmitted through various mechanisms including *Ornithodoros* ticks, oronasal contact, swill feeding and contaminated fomites. The World Organization for Animal Health designated ASF a notifiable disease due to its globally devastating economic impacts on the pig industry following the rapid expansion of the disease across eastern Europe and Asia from 2018. (*9*) To date, ASF has spread to over 19 countries in the Western Pacific Region. Feral pigs may be infected with ASF due to spillover from domestic piggeries, as observed in some parts of Asia and Europe. In Romania, for example, the proximity of feral pigs to domestic piggeries has been documented as the source of ASF. This suggests dual transmission between domestic and feral pig populations, leading to further amplification and geographic spread of the virus. The abundance, distribution and density of feral pigs are considered major factors driving the introduction of ASF into naïve areas.

FMD is caused by the FMD virus, a highly contagious transboundary viral disease of artiodactyls, with severe epidemics in susceptible animals affecting the socioeconomic livelihoods of affected communities through trade embargoes of livestock and their products. FMD is enzootic in Africa, Asia, the Middle East and South America. In vivo studies have demonstrated the persistence of the virus in the tonsils of feral pigs for over 30 days post-inoculation, which is characterized by the presence of vesicles in the oral cavity, interdigital spaces, and coronary bands of the hoof and udder. (*10*) Illegal trading and movement of animals from one place to another is considered a high-risk activity associated with the introduction of FMD into an area, as is swill feeding. Countries with large feral pig populations may have difficulty eradicating the disease, as it can circulate cryptically and may become endemic. Many countries in the Western Pacific Region that are FMD-free have instituted strict import policies and biosecurity measures at seaports and airports and have set up surveillance in livestock populations to prevent (or mitigate) possible incursions.

We suggest the establishment of surveillance programmes for monitoring the circulation of pathogens of zoonotic and veterinary concern among feral pigs, in addition to the expansion on the use of sentinel domestic pig herds as a system for the early detection of diseases that could potentially spill over to humans and other animals of economic importance. This would entail a joint regional One Health collaborative effort by all stakeholders from diverse fields, including risk communicators, modellers, ecologists, biosecurity experts, epidemiologists, virologists and anthropologists. Feral pig ethology should also be further studied to advance programmes for effective population control.
